# Analyzing pepsin degradation assay conditions used for allergenicity assessments to ensure that pepsin susceptible and pepsin resistant dietary proteins are distinguishable

**DOI:** 10.1371/journal.pone.0171926

**Published:** 2017-02-16

**Authors:** Rong Wang, Thomas C. Edrington, S. Bradley Storrs, Kathleen S. Crowley, Jason M. Ward, Thomas C. Lee, Zi L. Liu, Bin Li, Kevin C. Glenn

**Affiliations:** Monsanto Company, St. Louis, Missouri, United States of America; Iowa State University, UNITED STATES

## Abstract

The susceptibility of a dietary protein to proteolytic degradation by digestive enzymes, such as gastric pepsin, provides information on the likelihood of systemic exposure to a structurally intact and biologically active macromolecule, thus informing on the safety of proteins for human and animal consumption. Therefore, the purpose of standardized *in vitro* degradation studies that are performed during protein safety assessments is to distinguish whether proteins of interest are susceptible or resistant to pepsin degradation via a study design that enables study-to-study comparison. Attempting to assess pepsin degradation under a wide-range of possible physiological conditions poses a problem because of the lack of robust and consistent data collected under a large-range of sub-optimal conditions, which undermines the needs to harmonize *in vitro* degradation conditions. This report systematically compares the effects of pH, incubation time, and pepsin-to-substrate protein ratio on the relative degradation of five dietary proteins: three pepsin susceptible proteins [ribulose 1,5-bisphosphate carboxylase-oxygenase (Rubisco), horseradish peroxidase (HRP), hemoglobin (Hb)], and two pepsin resistant proteins [lipid transfer protein (LTP) and soybean trypsin inhibitor (STI)]. The results indicate that proteins susceptible to pepsin degradation are readily distinguishable from pepsin-resistant proteins when the reaction conditions are within the well-characterized optima for pepsin. The current standardized *in vitro* pepsin resistant assay with low pH and high pepsin-to-substrate ratio fits this purpose. Using non-optimal pH and/or pepsin-to-substrate protein ratios resulted in susceptible proteins no longer being reliably degraded by this stomach enzyme, which compromises the ability of this *in vitro* assay to distinguish between resistant and susceptible proteins and, therefore, no longer providing useful data to an overall weight-of-evidence approach to assessing safety of proteins.

## Introduction

The fate of most dietary proteins is degradation into small peptides and amino acids that are subsequently absorbed and predominantly used for new protein synthesis and energy [1]. This process is facilitated by the proteolytic enzyme pepsin, which is secreted by gastric chief cells as the precursor protein pepsinogen [2]. Coupled with the secretion of hydrochloric acid (HCl) by the parietal cells of the stomach lining, pepsinogen is autocatalytically activated to form pepsin, which is a broad-spectrum protease that preferentially hydrolyzes peptide bonds between the aromatic amino acids Phe, Trp and Tyr [3]. In general, the propensity for systemic absorption of any orally consumed protein/peptide is inversely proportional to the size of the molecule, with larger peptides not as readily absorbed as smaller ones [4].

The potential allergenicity of a protein or peptide has been linked with resistance to degradation by proteases, such as pepsin [5–7]. Many studies of the pepsin-mediated degradation of dietary proteins have been reported, with some confirming the hypothesis that proteins known to be allergenic are often resistant to pepsin degradation [8–10]. However, the correlation between degradation and loss of allergenic potential is not absolute. Some studies for selected dietary proteins have shown that *in vitro* pepsin resistance does not correlate to allergenic potential [11–14]. Study-to-study variations in the pH, purity and activity of pepsin, pepsin-to-substrate protein ratio, purity and conformation of the substrate protein, and incubation time have made it difficult to directly compare their results and determine if a true correlation between pepsin resistance and allergenicity exists [6, 15–20]. Consistent with this observation, an extensive review of *in vitro* digestibility studies identified a lack of harmonized test conditions as a confounding factor towards enabling comparison of results across studies [19]. This review reports that it is evident that no single *in vitro* model can fully represent all portions of *in vivo* digestion processes because human physiological conditions are highly complex and dynamic. For example, monitoring the intra-gastric pH in non-human primates found that the pH in the fasted stomach is around pH 2, but it then rises to a median peak of around pH 5 within 30 minutes after a meal before it gradually returns to the fasted state pH within several hours [21]. This pattern is similar to what has previously been observed in humans [22].

The present study is a systematic comparison of the effect of a wide range of pepsin degradation conditions on substrate protein degradation. The assay parameters utilized cover the range of conditions that have been used in the current *in vitro* pepsin resistant assay (low pH and high pepsin-to-substrate ratio) [23] and that may occur *in vivo*. The present work supports the conclusion that the experimental conditions that best fit the purpose of distinguishing pepsin-susceptible and pepsin-resistant dietary proteins are when the reaction conditions are within the well-characterized optima for pepsin.

## Materials and methods

### Enzymes and substrate proteins

Purified porcine pepsin (Cat. #P6887), ribulose 1,5-diphosphate carboxylase (Rubisco) (Cat. #R8000), hemoglobin (Hb) (Cat. #H2625), and soybean trypsin inhibitor (STI) (Cat. #T9003) were obtained from Sigma Aldrich (St Louis, MO). Horseradish peroxidase (HRP) (Cat. #31490) was purchased from Thermo Scientific (Grand Island, NY). Lipid transfer protein (LTP) was purified from corn at Monsanto Company (St. Louis, MO) using a combination of anion exchange (Q-Sepharose Fast Flow), cation exchange (S-Sepharose Fast Flow), and size exclusion chromatography (Sephacryl S-100). The sequence of purification steps used was based on a previously described procedure [24].

### Pepsin degradation

Prior to experimentation, the activity of the purchased pepsin (3404 U/mg of pepsin powder) at pH 1.2 was verified by degrading hemoglobin under a fixed condition at 37°C as previously described [25]. Pepsin stock solutions (2632 U/ml in solution) were prepared in 0.01 N HCl, 1% (w/v) NaCl and adjusted to a desired pH on the same day of an experiment. Purchased substrate proteins (HRP, Rubisco, Hb, and STI) were reconstituted in 1X PBS at > 1 mg/ml (w/v) on the same day of an experiment based on the data provided by the manufacturer. Total protein concentrations of freshly reconstituted protein solution were determined by Bradford assay, and subsequently diluted to 1 mg/ml. Internally produced substrate protein (LTP) was supplied in 50 mM sodium phosphate with 150 mM sodium chloride, pH 7.0. Its concentration was determined by amino acid analysis. All test proteins were also adjusted to the desired pH to match the respective pepsin stock solution immediately prior to pepsin degradation. The pepsin susceptible proteins (Rubisco, HRP, and Hb) were tested under 18 separate assay conditions; at six pH conditions (1.2, 2.0, 2.5, 3.0, 3.5, and 4.0) and three pepsin-to-substrate protein ratios (unit (U):μg), by mixing pH adjusted pepsin and protein solutions to the desired ratio (amount of pepsin:amount of substrate protein = 285 μl:75 μl, 57 μl:150 μl and 9.5 μl:250 μl for 10 U:1 μg, 1 U:1 μg and 0.1 U:1 μg, respectively). Pepsin and substrate protein mixtures were incubated in a 37°C water bath for durations of 0.5, 2, 5, 10, 20, 30, and 60 minutes before being quenched by the addition of 0.7 M Na_2_CO_3_ at 35% of the reaction volume. Each assay included three controls (pepsin only, substrate protein only, and quenched pepsin + substrate protein). In addition, samples and controls with two minute exposure to pepsin were collected for 24 assay conditions, including pH 5.0, pH 6.0, and three pepsin-to-substrate protein ratios for these three proteins. A minimum of triplicate assays for each test condition with pepsin susceptible proteins were carried out. The pepsin resistant proteins (LTP and STI) were only tested under representative conditions of pH 1.2 and 4.0 at the high pepsin-to-substrate protein ratio (10 U:1 μg). Assay controls, where either the substrate protein or the pepsin was omitted, were also subjected to identical experimental analysis.

### SDS–PAGE analysis

Samples and their associated controls from a minimum of three repeated assays taken at different time point of degradation reaction were subjected to SDS-PAGE utilizing NOVEX^™^ 10–20% (w/v) polyacrylamide Tricine SDS gels separated under constant voltage per the manufacturer’s instructions providing good separation of polypeptides between 2 and 20 kDa. NOVEX^™^ Mark 12^™^ unstained standards were used as molecular weight positional markers for each gel, with the smallest marker at 2.5 kDa. Following SDS-PAGE, proteins and polypeptides were visualized by Coomassie Blue staining and digitally imaged by a BIO-RAD GS-900 Densitometer (Hercules, CA). This scanner has a wide dynamic linear range of 0–3.4 OD and conducts a self-calibration before each gel scan using an internal standard certified to a National Institute of Standards and Technology (NIST)-traceable standard. The certified internal standard is calibrated biennially by the vendor to confirm accuracy and reproducibility following Good Laboratory Practices (GLP). The relative adjusted volume analysis tool of the BIO-RAD Image Lab 5.2.1 software was utilized to determine the percentage density of intact protein remaining relative to the no pepsin degradation control, which is used as the 100% value on those images. The sensitivity of Coomassie blue stain has a detection limit of 5 ng of BSA, as reported by the vendor (Expedeon Protein Solutions, distributed by Sigma-Aldrich) and verified experimentally by our lab. The relative adjusted volume of each data point was collected and presented in percentage from the gels for a minimum of triplicate assays at each test condition with pepsin susceptible proteins. Replicate data were analyzed to generate the standard deviations.

## Results

### pH dependence of degradation of proteins known to be pepsin susceptible

**Fig 1:**
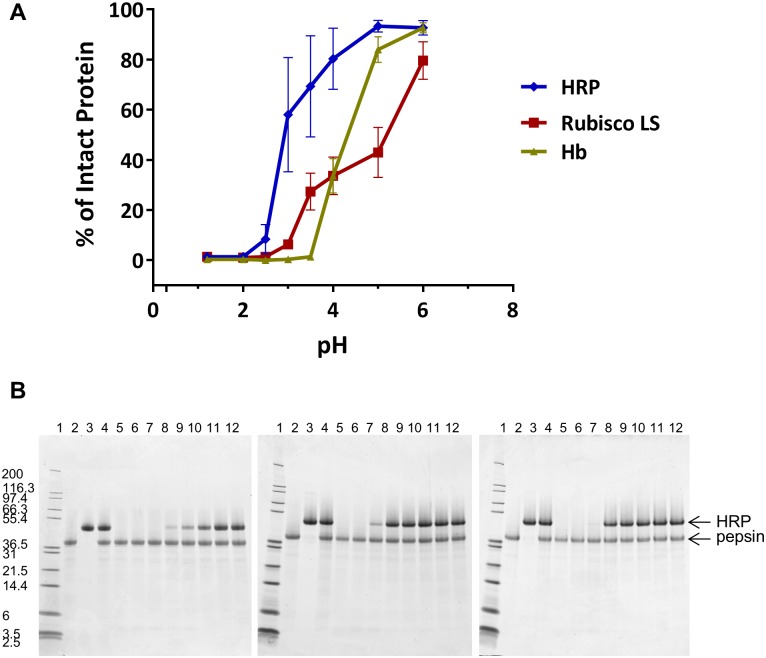
pH effect on pepsin degradation of three proteins (HRP, Rubisco, Hb) at 10 U:1 μg ratio for 2 minutes. Panel A: The amount of Coomassie Blue stained intact protein after exposure to pepsin for 2 minutes at each condition was quantified and is shown as a percentage relative to the amount of starting material. For Rubisco, only the large subunit (LS) was quantified as described in Results section. Panel B: Three gels from triplicate pepsin degradation assays with HRP. The gel lanes are: 1: MW, 2: Pepsin Only, 3: HRP protein Only, 4: 0 minute, Pepsin + HRP protein, 5–12: HRP exposed to pepsin for 2 min at pH 1.2, 2.0, 2.5, 3.0, 3.5, 4.0, 5.0, and 6.0, respectively.

To assess the influence of pH on the degradation of proteins by pepsin, three proteins known to be susceptible to pepsin degradation, ribulose 1,5-bisphosphate carboxylase-oxygenase (Rubisco), horseradish peroxidase (HRP), and hemoglobin (Hb) [23, 26–28], were incubated at a constant pepsin-to-substrate protein ratio (10 U:1 μg substrate protein) following a standardized protocol [23] but using a range of pH values from 1.2–6.0 for a duration of 2 minutes (Fig 1, S1 File). Because the large and small subunits of Rubisco disassociate during SDS-PAGE analysis, the protein band corresponding to the Rubisco large subunit (LS) was utilized for this analysis to avoid ambiguity between a degradation product and the Rubisco small subunit. The results showed that pepsin degraded ≥ 99% of the intact forms of HRP protein at pH ≤ 2.0, of Rubisco LS protein at pH ≤ 2.5, and of Hb proteins at pH ≤ 3.5. Therefore, susceptibility to pepsin hydrolysis of these three proteins was consistent at a pH ≤ 2.0. Variability between the extent of pepsin’s ability to degrade the intact forms of these three proteins was observed at pH values > 2.0. There was little meaningful pepsin-mediated degradation of these three proteins at pH 6.0.

### pH and pepsin-to-substrate ratio dependence of degradation of proteins known to be pepsin susceptible

**Fig 2:**
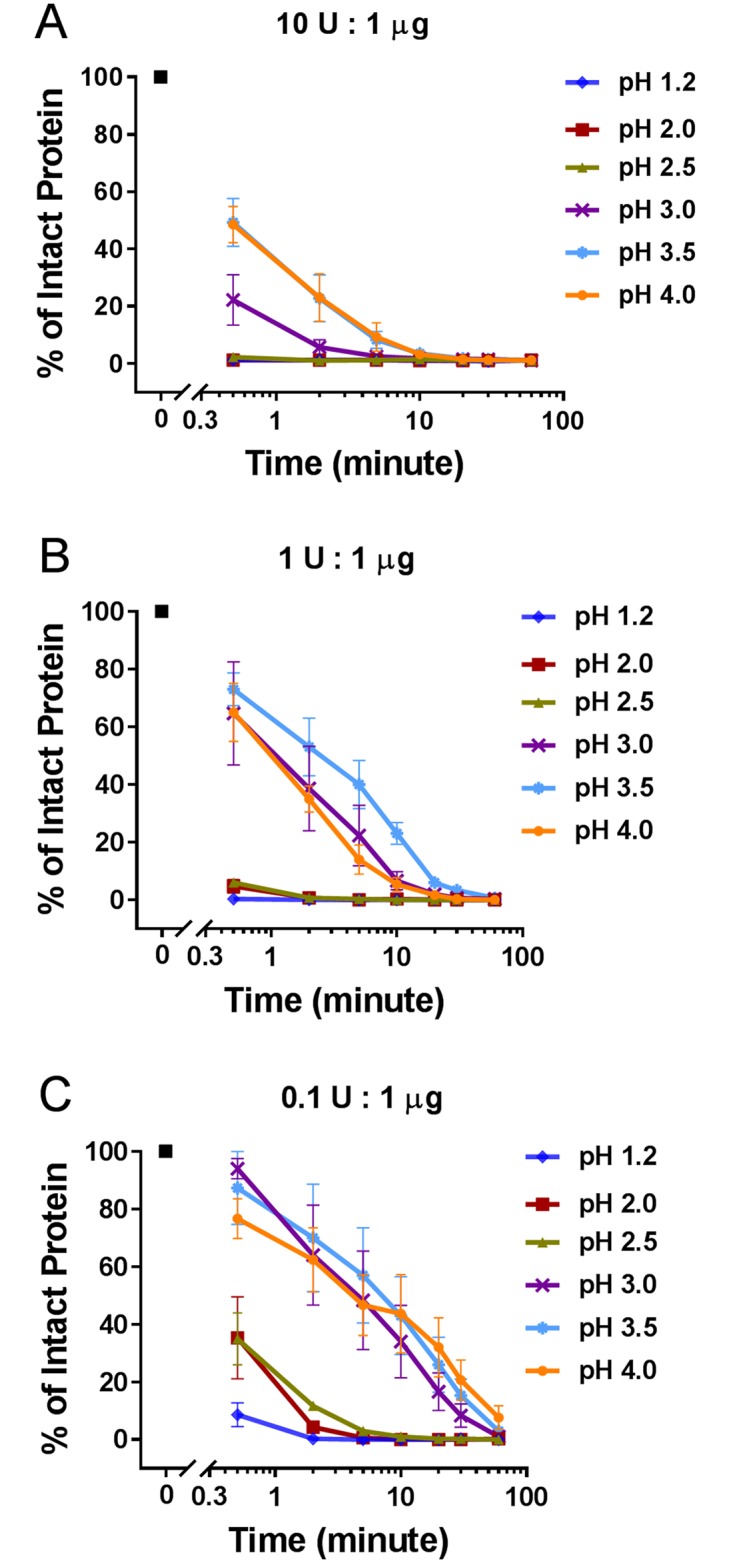
Effect of pepsin-to-substrate protein ratio and time on pepsin degradation of Rubisco LS at six different pHs. The amount of Coomassie Blue stained intact protein at each condition was quantified and is shown as a percentage relative to the amount of starting material. A) 10 U:1 μg; B) 1 U:1 μg; C) 0.1 U:1 μg.

Based upon the observation that ≥ 34% of the intact Rubisco LS was degraded after 2 minutes when pH is ≤ 4.0, the range of pH 1.2–4.0 was used to characterize the effect of the ratio of pepsin-to-substrate protein (10 U:1 μg, 1 U:1 μg and 0.1 U:1 μg) on the pepsin degradation of these proteins. The first set of experiments focused on assessing the degradation of the Rubisco LS at three ratios of pepsin. The results in Fig 2 (S2 File) indicate that intact Rubisco LS was rapidly (≤ 2 minutes) and completely (≥ 95%) degraded at pepsin-to-substrate protein ratios of 10 U:1 μg (Fig 2A, S2 File) and 1 U:1 μg (Fig 2B, S2 File) between pH 1.2 to 2.5. By comparison, at a pepsin-to-substrate protein ratio of 0.1 U:1 μg (Fig 2C, S2 File) with pH 2.0 and 2.5, nearly 4% and 12% of the intact Rubisco LS, respectively, was still present after 2 minutes. At pH values ≥ 3.0, the decreased efficacy of pepsin to degrade substrate proteins becomes even more evident as the ratio of pepsin-to-substrate protein is decreased, which is consistent with the results at 10 U:1 μg depicted in Fig 1 (S1 File). At pH 3.0–4.0, ≥ 64% of intact Rubisco LS was still observable after 2 minutes of incubation at a pepsin-to-substrate protein ratio of 0.1 U pepsin to 1 μg Rubisco (Fig 2C, S2 File). Under all the pepsin-to-substrate protein ratios and pH conditions tested, except for the 0.1 U pepsin to 1 μg Rubisco ratio at pH 4.0, degradation of 97% of the intact Rubisco LS was observed after 60 minutes.

**Fig 3:**
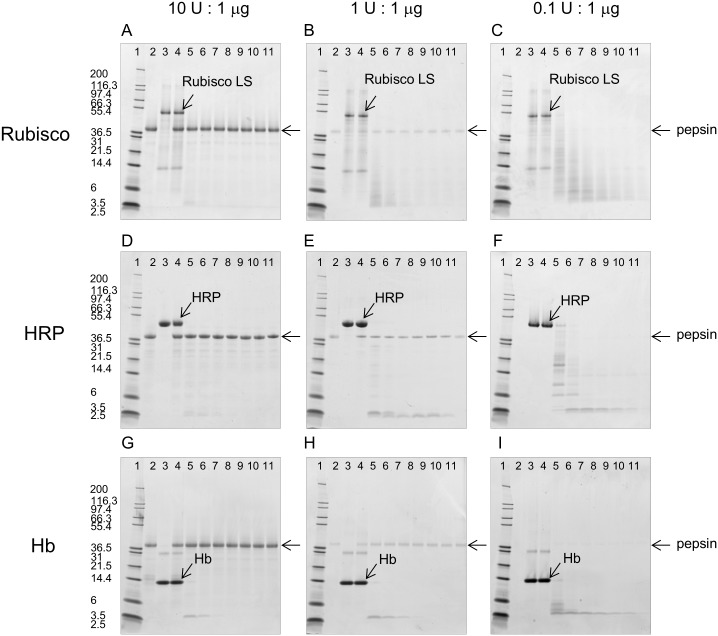
SDS-PAGE analysis of pepsin degradation of HRP, Rubisco, and Hb at pH 1.2 and three pepsin-to-substrate protein ratios. 1 μg of substrate protein, based upon the pre-degradation concentration, was loaded in each well. For all figure panels, the gel lanes are: 1: MW, 2: Pepsin Only, 3: Substrate Protein Only, 4: 0 minute, Pepsin + Substrate Protein, 5–11: 0.5, 2, 5, 10, 20, 30, 60 minute(s), respectively, of Pepsin + Substrate Protein. Figure Panels: A) Rubisco, 10 U:1 μg; B) Rubisco, 1 U:1 μg; C) Rubisco, 0.1 U:1 μg; D) HRP, 10 U:1 μg; E) HRP, 1 U:1 μg; F) HRP, 0.1 U:1 μg; G) Hb, 10 U:1 μg; H) Hb, 1 U:1 μg; I) Hb, 0.1 U:1 μg.

The effect of three pepsin-to-substrate ratios on degradation of the three substrate proteins (Rubisco, HRP and Hb) by pepsin at pH 1.2 was analyzed by SDS-PAGE and is shown in Fig 3. The intensity of the stained band corresponding to pepsin decreased as the amount of pepsin decreased 100-fold from 10 Units to 0.1 Unit. For all three substrate proteins at this low pH value 1.2, the intact form was rapidly degraded within 2 minutes at all pepsin-to-substrate protein ratios. The one exception was a faint band corresponding to the intact form of HRP observed at the 0.1 U pepsin to 1 μg HRP ratio. However, this band was no longer visible after the 2 minute incubation time with pepsin at this low ratio. In contrast, the degradation fragments of each substrate protein, and the duration of the persistence of these fragments, increased as the pepsin-to-substrate protein ratio decreased. For example, there were significantly more degradation fragments of HRP present in the low pepsin-to-substrate protein ratio (0.1 U:1 μg) compared to the high pepsin-to-substrate protein ratio (10 U:1 μg).

**Fig 4:**
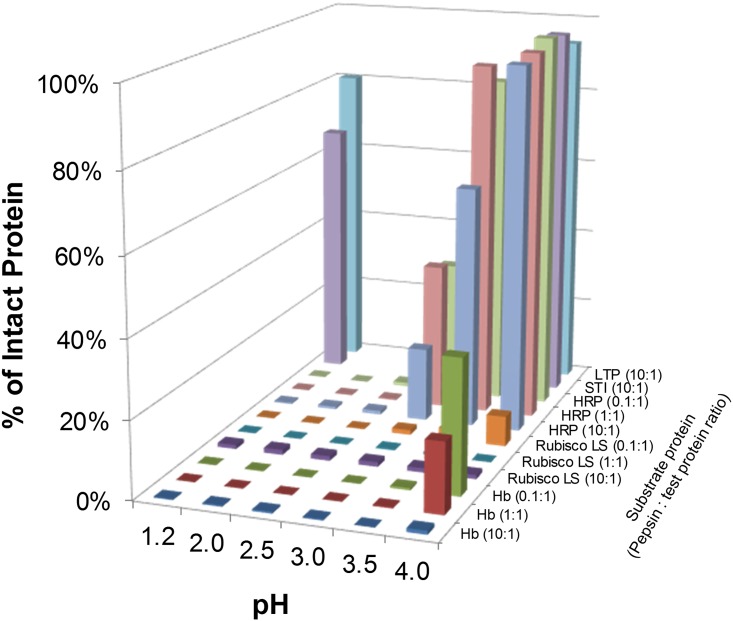
Quantification of degradation of HRP, Rubisco LS, Hb, STI and LTP at various pH conditions and pepsin-to-substrate protein ratios after 60-minute incubation with pepsin. The amount of Coomassie Blue stained intact protein at each condition was quantified and is shown as a percentage relative to the amount of starting material.

The relationship between pepsin-to-substrate protein ratio and pH on the efficacy of the pepsin-mediated degradation of Rubisco LS, HRP, Hb, lipid transfer protein (LTP) and soybean trypsin inhibitor (STI) after 60 minutes is depicted in Fig 4 (S3 File). The results indicate that pepsin degrades ≥ 97% of Hb and Rubisco LS at all tested pepsin-to-substrate ratios and pH levels except for pH 4.0. At pH 4.0 and pepsin-to-substrate protein ratios of 1 U:1 μg and 0.1 U:1 μg, 0% and 8% of intact Rubisco LS while, at the same ratio, 18% and 35% of intact Hb were still observed after 60 minutes. By comparison, the results indicate that pepsin degradation of HRP is relatively more dependent than the Rubisco LS and Hb on pH and the enzyme to substrate ratio. At pH 3.0, 19–35% of intact HRP remained after 60 minutes at all three pepsin-to-substrate ratios. At 3.5 and 4.0 pH, 63% to nearly 99% of intact HRP was not degraded by pepsin after 60 minutes. By comparison, even under the highest tested pepsin-to-substrate ratio (10 U: 1 μg), both STI and LTP, each known to be resistant to pepsin, were virtually unaffected by pepsin at the two extremes of pH (1.2 and 4.0) as shown (Fig 4, S3 File).

### Comparison of pepsin degradation of proteins known to be susceptible or resistant to pepsin

**Fig 5:**
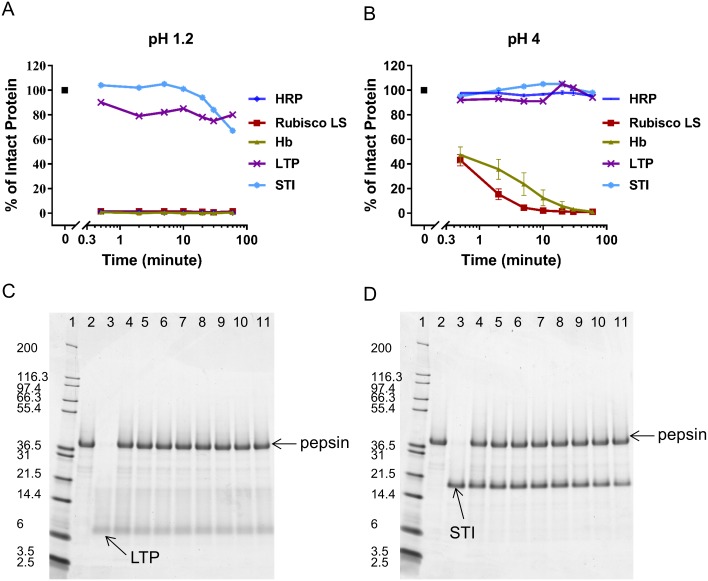
Comparison of pepsin degradation of five substrate proteins (HRP, Rubisco LS, Hb, STI, and LTP). On Panel A and Panel B, the amount of Coomassie Blue stained intact protein at each condition was quantified and is shown as a percentage relative to the amount of starting material. Panel A is at 10 U pepsin to 1 μg substrate protein and pH 1.2. Panel B is at 10 U pepsin to 1 μg substrate protein and pH 4. Panels C and D are SDS-PAGE analysis of pepsin degradation of LTP and STI, respectively, at pH 1.2 and 10 U pepsin to 1 μg substrate protein over time (0.5 to 60 minute(s)). The gel lanes are: 1: MW, 2: Pepsin Only, 3: Substrate Protein Only, 4: 0 minute: Pepsin + Substrate Protein, 5–11: 0.5, 2, 5, 10, 20, 30, 60 minute(s), respectively, of Pepsin + Substrate Protein.

To further compare the effect of pepsin degradation of LTP and STI with the three pepsin susceptible proteins (Rubisco, HRP and Hb), a time course of experiments was conducted. When assayed under a standardized pepsin degradation condition of pH 1.2 and 10 U pepsin to 1 μg substrate protein, Rubisco LS, HRP and Hb were all degraded by > 98% within 0.5 minute. In contrast, the intact forms of LTP and STI were relatively stable for over 60 minutes (Fig 5A, S4 File). At pH 4.0 and 10 U pepsin to 1 μg substrate protein, the amount of Rubisco LS and Hb degradation was reduced, and by 60 minutes the intact form of each protein was fully degraded (Fig 5B, S4 File). However, ≥ 96% of the intact HRP protein persisted throughout the experiment incubation time under these sub-optimal pepsin degradation conditions (Fig 5B, S4 File). Under both enzymatic reaction conditions, low pH/high amount of pepsin and high pH/low amount of pepsin, > 67% of LTP and STI remained intact over 60 minutes (Fig 5A and 5B, S4 File). Further analysis by SDS-PAGE of the LTP and STI reaction mixtures at pH 1.2 and 10 U pepsin to 1 μg substrate protein revealed no potential degradation fragments for up to 60 minutes (Fig 5C and 5D, respectively).

## Discussion

The physiological conditions of protein digestion are highly complex and constantly changing such that no single *in vitro* model can fully represent all portions of *in vivo* digestion processes [19]. The present studies systematically compared the effect of several independent variables that are physiologically and biochemically relevant to gastric pepsin degradation on the ability to reproducibly distinguish between pepsin susceptible and resistant proteins. By understanding the relationship between experimental variables known to affect pepsin proteolytic activity, it is possible to determine the optimal conditions that biochemically measure a protein’s overall physicochemical stability in the presence of pepsin. A well-defined *in vitro* method can be useful to ensure accurate, reproducible and biologically meaningful results are obtained that enable the comparison of pepsin susceptibility across various substrate dietary proteins [15, 23, 29]. The independent variables that were controlled in the present study included selection of substrate proteins that are biochemically representative of both pepsin susceptible and pepsin resistant dietary proteins, pH, the ratio of pepsin-to-substrate protein, and reaction time.

### Substrate protein selection

The proteins selected for this analysis included three proteins known to be susceptible to pepsin degradation [ribulose 1,5-bisphosphate carboxylase-oxygenase large subunit (Rubisco LS), horseradish peroxidase (HRP), hemoglobin (Hb)] and two known to be pepsin-resistant [lipid transfer protein (LTP) and soybean trypsin inhibitor (STI)]. All five of these substrate proteins have been well characterized [30–35]. Selection of the substrate proteins focused on identifying a set of substrates with a diversity of physiochemical properties that could affect pepsin hydrolysis. For candidate proteins, amino acid sequences from the National Center for Biotechnology Information (NCBI) were used to calculate the theoretical *pI*, molecular weight, intramolecular disulfide bonds and number of aromatic amino acids (Table 1). The theoretical *pIs* ranged from a low of 4.91 for STI [36] to a high of 9.47 for LTP [37], and molecular weights ranged from 9 kDa for LTP to ~500 kDa for Rubisco (taking into account that Rubisco contains 8 large subunits at ~ 52 kDa each and 8 small subunits at ~13 kDa each) [38]. All substrate proteins (Table 1) contained different levels of disulfide bonds from zero for Hb [39] to four for LTP and HRP [37, 40]. The number of aromatic amino acids ranged from 2% for LTP (2 out of the 93 amino acids) to 10% for Rubisco LS (47 out of 475 amino acids). The prevalence of aromatic amino acids is critical because pepsin is a broad-spectrum protease that preferentially hydrolyzes peptide bonds between the aromatic amino acids Phe, Trp and Tyr [3]. The percentage of pepsin cleavage sites also varied for the substrate proteins. For example, the Rubisco LS contains 474 peptide bonds, with the maximum number of theoretical cleavage sites for pepsin calculated to be 122 (http://web.expasy.org/peptide_cutter/). These cleavage sites represent approximately 26% of the total peptide bonds found in the Rubisco LS. In contrast, the maximum theoretical cleavages of lipid transfer protein (LTP) are only eight out of a total of 92 peptide bonds (http://web.expasy.org/peptide_cutter/), which account for approximately 9% of total peptide bonds.

**Table 1:** Five test proteins represent diverse characteristics of physiochemical properties that could affect pepsin hydrolysis.

Substrate Protein Name	Molecular Weight (kDa)^1^	Theoretical pI^1^	Number of Disulfide Bonds	Number of Aromatic Amino Acids in Total (Percent of Total)^1^	Estimated Number of Cleavage Sites^2^
**Rubisco Large Subunit (LS)**^3^	210.7	6.46	2	47/475 (10%)	122
**Horseradish Peroxidase (HRP)**^4^	34.05	6.63	4	26/309 (8%)	81
**Hemoglobin (Hb) Subunit a**^5^	15.04	9.26	0	10/141 (7%)	40
**Hemoglobin (Hb) Subunit b**^6^	16.03	7.98	0	12/146 (8%)	42
**Soybean Trypsin Inhibitor (STI)**^7^	20.10	4.91	2	15/181 (8%)	55
**Lipid Transfer Protein (LTP)**^8^	9.05	9.47	4	2/93 (2%)	8

^1^ Calculated based on amino acid sequences using CLC Genomic Workbench (QIAGEN).

^2^ Estimated using PeptideCutter (http://web.expasy.org/peptide_cutter/).

^3^ NCBI accession number: NP_054944

^4^ NCBI accession number: CAA00083

^5^ NCBI accession number: 2PGH_A

^6^ NCBI accession number: 2PGH_B

^7^ NCBI accession number: 1BA7_A

^8^ NCBI accession number: AAA33493

### Effect of pH on substrate hydrolysis by pepsin

The results detailed in Fig 1 (S1 File) indicate that between pH 1.2–2.5 pepsin effectively and rapidly degraded three substrate proteins (Rubisco, HRP, and Hb) that are known to be susceptible to pepsin degradation. This observation is as expected since the optimal pH for pepsin’s proteolytic activity is 1.6, and is consistent with the observation that pepsin optimally degrades dietary proteins between a pH range of 1.2 to 2.5 [26, 27]. Pepsin is an aspartic protease and its activity is directly dependent on the pH of the solution environment [41]. At pH values >2.5, pepsin activity begins to decline, with incomplete degradation of the three susceptible substrate proteins by pepsin being observed. Similar observations were previously reported for a number of pepsin susceptible proteins from whey and rice [10, 42]. The variability in the extent of degradation of each substrate protein by pepsin at pH values >2.5 could be attributed to a number of factors including inter-analyst variability, batch-to-batch gel variability, and staining solution variability, etc. It is also likely influenced by the unique physiochemical properties of each of the substrate proteins in different pH environments. For example, HRP is quite stable in neutral conditions but has been reported to lose its structural stability below pH 4.5 and appears to have an acid-induced unfolding at pH 3.0 [30], which corresponds to the observed increase in susceptibility to pepsin degradation of HRP at approximately pH 3.0. Therefore, in addition to pepsin-mediated peptide bond hydrolysis being most predictable when pH ≤ 2.5, limiting the variability of pH can also facilitate the ability to compare the relative susceptibility of different substrate proteins for pepsin degradation by standardizing the acid denaturation conditions [43].

### Effect of the pepsin-to-substrate protein ratio on substrate degradation

Previous reports have indicated that, under conditions that attempt to be more physiologically relevant, degradation of substrate proteins is slower or less complete because those conditions are sub-optimal for pepsin (e.g., higher pH and/or lower ratios of pepsin-to-substrate protein) [10, 44]. To further characterize the effect of the ratio of pepsin-to-substrate protein on degradation of these same three substrate proteins (Rubisco, HRP, and Hb), three ratios were used: 10 U:1 μg, 1 U:1 μg and 0.1 U:1 μg. Each of these three ratios was tested at pH ranging from 1.2–4.0 with multiple incubation times or at pH 5.0 and 6.0 with a single incubation time. Given that the specific activity of pepsin can vary from preparation to preparation, the enzyme to substrate ratio was based on the units of pepsin activity and not the amount of pepsin protein being used. The results indicated that the extent of degradation of substrate proteins was directly proportional to the ratio of units of pepsin activity to substrate protein (Figs 2 to 5). The higher pepsin-to-substrate protein ratio (10 U:1 μg) resulted in a lower percentage of intact protein at all incubation times and pH values, compared with the middle (1 U:1 μg) or low ratio (0.1 U:1 μg). Nearly complete degradation of the three substrate proteins after two minutes was limited to an assay condition at the 10 U:1 μg ratio, which is the standardized and commonly applied condition previously established [23]. In contrast, two substrate proteins that are known to be resistant to pepsin degradation, LTP and STI, exhibited pepsin-resistance at the higher pepsin-to-substrate protein ratio (10 U:1 μg) regardless of the test pH (Figs 4 and 5). Thus, not only does the pH of an assay condition greatly affect the ability of pepsin to degrade substrate proteins, but also the ratio of pepsin-to-substrate protein can substantially influence the assessment of pepsin’s ability to degrade a substrate protein.

## Conclusions

Enzymes have optimal conditions that are well characterized. Changing those conditions will have a detrimental effect on the enzyme activity. This is well known in traditional biochemistry, so the slow or incomplete degradation from higher pH and low ratio of pepsin-to-substrate protein that was observed in this study is not surprising. Raising the pH and/or lowering the pepsin-to-substrate protein ratio increased the number of observed fragments and/or increased duration of persistence of intact forms of pepsin susceptible proteins.

The standardized and commonly applied *in vitro* pepsin resistance assay can distinguish between pepsin susceptible and resistant proteins as it is currently validated [23]. Stability to pepsin is one of several factors that provides information on the likelihood of exposure to an active and intact protein as part of the weight-of-evidence assessment of the safety of a protein even though the correlation between the allergenicity and pepsin resistant is not absolute. The pepsin digestion assay provides useful information to compare the possibility of exposure to the intact form of one protein, to that of another protein. The results in the present study show that pepsin susceptible proteins are readily distinguishable from pepsin resistant proteins when the reaction conditions are within the pH optima well known for pepsin and effective pepsin-to-substrate protein ratio. The use of non-optimal pH and/or pepsin-to-substrate protein ratio conditions resulted in pepsin susceptible proteins no longer being reliably degraded by this stomach enzyme. Therefore, the utility of this *in vitro* assay to test substrate protein degradation by pepsin is limited when the experimental conditions are sub-optimal for the enzyme.

Utilizing an *in vitro* pepsin assay performed under non-optimal and varying conditions to determine the susceptibility of dietary proteins to pepsin degradation does not provide any additional information on the safety of the protein, nor the potential for the protein to be an allergen, compared to what can be obtained from performing the assay under the previously established standardized conditions [23]. Indeed, the results presented herein demonstrate the converse, in that utilizing non-optimal and varying conditions to assess a protein’s susceptibility to pepsin degradation diminishes the ability to obtain clear and consistent results that can be directly compared across multiple studies. Thus, the standardized and commonly applied *in vitro* pepsin degradation assay is sufficient as part of the weight of evidence to support protein safety. The results of this study support the conclusion that assays, such as this assessment of the susceptibility of a protein to be digested by pepsin, require well-defined experimental conditions that best fit the purpose of the assay. This enables the assay to provide interpretable data as part of the weight-of-evidence approach used to assess safety of proteins introduced into GM crops.

## Supporting information

S1 File Supporting information for Fig 1. (PDF)

S2 File Supporting information for Fig 2. (PDF)

S3 File Supporting information for Fig 4. (PDF)

S4 File Supporting information for Fig 5. (PDF)
