# Supplementary material for: Analyzing pepsin degradation assay conditions used for allergenicity assessments to ensure that pepsin susceptible and pepsin resistant dietary proteins are distinguishable
Source: PLoS One. 2017 Feb 16;12(2):e0171926. doi: 10.1371/journal.pone.0171926 (PMC5312868; doi:10.1371/journal.pone.0171926)
Supplement: S1 File — (PDF) [file pone.0171926.s001.pdf]

Supporting Information

Fig 1. pH effect on pepsin degradation of three proteins (HRP, Rubisco, Hb) at 10 U:1 μg ratio for 2 minutes. Panel A: The amount of Coomassie Blue stained intact protein after exposure to pepsin for 2 minutes at each condition was quantified and is shown as a percentage relative to the amount of starting material. For Rubisco, only the large subunit (LS) was quantified as described in Results section. Panel B: Three gels from triplicate pepsin degradation assays with HRP. The gel lanes are: 1: MW, 2: Pepsin Only, 3: HRP protein Only, 4: 0 minute, Pepsin + HRP protein, 5– 12: HRP exposed to pepsin for 2 min at pH 1.2, 2.0, 2.5, 3.0, 3.5, 4.0, 5.0, and 6.0, respectively.

The average and standard deviation of relative adjusted volume from densitometric analysis for the three pepsin susceptible proteins (HRP, Rubisco LS, Hb) are listed below. The data were used to plot the line graph.

Rubisco

	Average of Relative Adjusted Volume from Densitometry								STDEV of Relative Adjusted Volume from Densitometry							
E:S*	pH 1.2	pH 2	pH 2.5	pH 3	pH 3.5	pH 4	pH 5	pH 6	pH 1.2	pH 2	pH 2.5	pH 3	pH 3.5	pH 4	pH 5	pH 6
10:1	1%	1%	1%	7%	27%	34%	43%	76%	0%	0%	1%	2%	13%	13%	17%	10%
1:1	0%	0%	1%	11%	28%	35%	49%	91%	1%	1%	0%	1%	3%	6%	6%	2%
1:10	1%	1%	13%	45%	61%	66%	72%	90%	0%	0%	6%	8%	4%	3%	7%	7%

HRP

	Average of Relative Adjusted Volume from Densitometry								STDEV of Relative Adjusted Volume from Densitometry							
E:S*	pH 1.2	pH 2	pH 2.5	pH 3	pH 3.5	pH 4	pH 5	pH 6	pH 1.2	pH 2	pH 2.5	pH 3	pH 3.5	pH 4	pH 5	pH 6
10:1	1%	1%	8%	58%	69%	81%	93%	93%	1%	0%	10%	39%	35%	21%	4%	5%
1:1	0%	0%	14%	80%	94%	94%	99%	99%	1%	0%	16%	20%	11%	7%	2%	5%
1:10	1%	1%	15%	78%	83%	97%	100%	98%	1%	1%	13%	19%	17%	5%	1%	1%

Hb

	Average of Relative Adjusted Volume from Densitometry								STDEV of Relative Adjusted Volume from Densitometry							
E:S*	pH 1.2	pH 2	pH 2.5	pH 3	pH 3.5	pH 4	pH 5	pH 6	pH 1.2	pH 2	pH 2.5	pH 3	pH 3.5	pH 4	pH 5	pH 6
10:1	0%	0%	0%	0%	1%	34%	84%	93%	0%	0%	0%	0%	0%	12%	9%	4%
1:1	1%	0%	0%	0%	2%	47%	92%	101%	1%	0%	0%	0%	1%	12%	3%	3%
1:10	0%	0%	0%	1%	5%	54%	96%	97%	0%	0%	0%	0%	1%	9%	4%	4%

* E:S refers to enzyme and substrate protein ratio at unit of pepsin per μg of substrate protein.
