# Supplementary material for: Analyzing pepsin degradation assay conditions used for allergenicity assessments to ensure that pepsin susceptible and pepsin resistant dietary proteins are distinguishable
Source: PLoS One. 2017 Feb 16;12(2):e0171926. doi: 10.1371/journal.pone.0171926 (PMC5312868; doi:10.1371/journal.pone.0171926)
Supplement: S2 File — (PDF) [file pone.0171926.s002.pdf]

Fig 2. Effect of pepsin-to-substrate protein ratio and time on pepsin degradation of Rubisco LS at six different pHs. The amount of Coomassie Blue stained intact protein at each condition was quantified and is shown as a percentage relative to the amount of starting material. A) 10 U:1 μg; B) 1 U:1μg; C) 0.1 U:1 μg.

The average and standard deviation of relative adjusted volume from densitometric analysis for the pepsin susceptible Rubisco LS protein are listed below. The data were used to plot the line graphs.

	Average of Relative Adjusted Volume from Densitometry (E:S*=10:1)						STDEV of Relative Adjusted Volume from Densitometry(E:S*=10:1)					
Minutes	pH 1.2	pH 2	pH 2.5	pH 3	pH 3.5	pH 4	pH 1.2	pH 2	pH 2.5	pH 3	pH 3.5	pH 4
0.5	1%	1%	2%	22%	49%	48%	1%	1%	1%	18%	17%	13%
2	1%	1%	1%	6%	23%	23%	0%	0%	0%	5%	16%	16%
5	1%	2%	1%	2%	8%	9%	0%	0%	0%	2%	6%	10%
10	1%	1%	1%	2%	4%	3%	0%	0%	0%	1%	2%	3%
20	1%	1%	1%	1%	2%	2%	0%	1%	0%	0%	0%	1%
30	1%	1%	1%	1%	2%	1%	0%	1%	0%	0%	0%	0%
60	1%	1%	1%	1%	1%	1%	0%	0%	0%	0%	1%	1%

	Average of Relative Adjusted Volume from Densitometry (E:S*=1:1)						STDEV of Relative Adjusted Volume from Densitometry (E:S*=1:1)					
Minutes	pH 1.2	pH 2	pH 2.5	pH 3	pH 3.5	pH 4	pH 1.2	pH 2	pH 2.5	pH 3	pH 3.5	pH 4
0.5	0%	5%	6%	65%	73%	65%	1%	4%	1%	31%	10%	17%
2	0%	1%	1%	39%	53%	35%	0%	0%	0%	26%	17%	8%
5	0%	0%	1%	22%	40%	14%	0%	0%	1%	18%	14%	9%
10	0%	0%	0%	7%	23%	6%	0%	0%	0%	6%	6%	4%
20	0%	0%	0%	2%	6%	2%	0%	0%	0%	2%	1%	1%
30	0%	0%	0%	1%	3%	1%	0%	0%	0%	0%	1%	0%
60	0%	0%	0%	0%	0%	0%	0%	0%	0%	0%	0%	0%

	Average of Relative Adjusted Volume from Densitometry (E:S*=0.1:1)						STDEV of Relative Adjusted Volume from Densitometry (E:S*=0.1:1)					
Minutes	pH 1.2	pH 2	pH 2.5	pH 3	pH 3.5	pH 4	pH 1.2	pH 2	pH 2.5	pH 3	pH 3.5	pH 4
0.5	9%	35%	35%	94%	87%	77%	7%	25%	15%	6%	22%	11%
2	1%	4%	12%	64%	70%	62%	0%	3%	3%	30%	32%	19%
5	0%	1%	3%	48%	57%	47%	0%	1%	1%	29%	28%	18%
10	3%	0%	1%	34%	43%	44%	4%	0%	1%	21%	23%	23%
20	0%	0%	0%	17%	26%	32%	0%	0%	0%	12%	16%	17%
30	0%	0%	0%	8%	15%	20%	0%	0%	0%	7%	10%	12%
60	0%	0%	0%	1%	3%	8%	0%	0%	0%	1%	2%	7%

* E:S refers to enzyme and substrate protein ratio at unit of pepsin per μg of substrate protein.
