# Supplementary material for: Analyzing pepsin degradation assay conditions used for allergenicity assessments to ensure that pepsin susceptible and pepsin resistant dietary proteins are distinguishable
Source: PLoS One. 2017 Feb 16;12(2):e0171926. doi: 10.1371/journal.pone.0171926 (PMC5312868; doi:10.1371/journal.pone.0171926)
Supplement: S3 File — (PDF) [file pone.0171926.s003.pdf]

Supporting Information

Fig 4. Quantification of degradation of HRP, Rubisco LS, Hb, STI and LTP at various pH conditions and pepsin-to-substrate protein ratios after 60-minute incubation with pepsin. The amount of Coomassie Blue stained intact protein at each condition was quantified and is shown as a percentage relative to the amount of starting material.

The average and standard deviation of relative adjusted volume from densitometric analysis for the three pepsin susceptible proteins (HRP, Rubisco LS, Hb) are listed below as well as the relative adjusted volume for the two pepsin resistant proteins (LTP and STI). The data were used to plot the bar graph.

HRP

	Average of Relative Adjusted Volume from Densitometry (E:S*=10:1)						STDEV of Relative Adjusted Volume from Densitometry (E:S*=10:1)					
Minutes	pH 1.2	pH 2	pH 2.5	pH 3	pH 3.5	pH 4	pH 1.2	pH 2	pH 2.5	pH 3	pH 3.5	pH 4
60	0%	1%	1%	19%	63%	96%	0%	1%	1%	3%	33%	1%

	Average of Relative Adjusted Volume from Densitometry (E:S*=1:1)						STDEV of Relative Adjusted Volume from Densitometry (E:S*=1:1)					
Minutes	pH 1.2	pH 2	pH 2.5	pH 3	pH 3.5	pH 4	pH 1.2	pH 2	pH 2.5	pH 3	pH 3.5	pH 4
60	0%	0%	0%	38%	93%	97%	0%	0%	0%	9%	4%	4%

	Average of Relative Adjusted Volume from Densitometry (E:S*=0.1:1)						STDEV of Relative Adjusted Volume from Densitometry (E:S*=0.1:1)					
Minutes	pH 1.2	pH 2	pH 2.5	pH 3	pH 3.5	pH 4	pH 1.2	pH 2	pH 2.5	pH 3	pH 3.5	pH 4
60	0%	0%	1%	35%	87%	99%	0%	0%	1%	4%	5%	2%

Rubisco LS

	Average of Relative Adjusted Volume from Densitometry (E:S*=10:1)						STDEV of Relative Adjusted Volume from Densitometry (E:S*=10:1)					
Minutes	pH 1.2	pH 2	pH 2.5	pH 3	pH 3.5	pH 4	pH 1.2	pH 2	pH 2.5	pH 3	pH 3.5	pH 4
60	1%	1%	1%	1%	1%	1%	0%	0%	0%	0%	1%	1%

	Average of Relative Adjusted Volume from Densitometry (E:S*=1:1)						STDEV of Relative Adjusted Volume from Densitometry (E:S*=1:1)					
Minutes	pH 1.2	pH 2	pH 2.5	pH 3	pH 3.5	pH 4	pH 1.2	pH 2	pH 2.5	pH 3	pH 3.5	pH 4
60	0%	0%	0%	0%	0%	0%	0%	0%	0%	0%	0%	0%

	Average of Relative Adjusted Volume from Densitometry (E:S*=0.1:1)						STDEV of Relative Adjusted Volume from Densitometry (E:S*=0.1:1)					
Minutes	pH 1.2	pH 2	pH 2.5	pH 3	pH 3.5	pH 4	pH 1.2	pH 2	pH 2.5	pH 3	pH 3.5	pH 4
60	0%	0%	0%	1%	3%	8%	0%	0%	0%	1%	2%	7%

Hb

	Average of Relative Adjusted Volume from Densitometry (E:S*=10:1)						STDEV of Relative Adjusted Volume from Densitometry (E:S*=10:1)					
Minutes	pH 1.2	pH 2	pH 2.5	pH 3	pH 3.5	pH 4	pH 1.2	pH 2	pH 2.5	pH 3	pH 3.5	pH 4
60	0%	0%	0%	0%	0%	1%	0%	0%	0%	0%	0%	1%

	Average of Relative Adjusted Volume from Densitometry (E:S*=1:1)						STDEV of Relative Adjusted Volume from Densitometry (E:S*=1:1)					
Minutes	pH 1.2	pH 2	pH 2.5	pH 3	pH 3.5	pH 4	pH 1.2	pH 2	pH 2.5	pH 3	pH 3.5	pH 4
60	0%	0%	0%	0%	0%	18%	0%	0%	0%	0%	0%	10%

	Average of Relative Adjusted Volume from Densitometry (E:S*=0.1:1)						STDEV of Relative Adjusted Volume from Densitometry (E:S*=0.1:1)					
Minutes	pH 1.2	pH 2	pH 2.5	pH 3	pH 3.5	pH 4	pH 1.2	pH 2	pH 2.5	pH 3	pH 3.5	pH 4
60	0%	0%	0%	0%	1%	35%	0%	0%	0%	0%	1%	20%

LTP

	Relative Adjusted Volume from Densitometry (E:S*=10:1)
Minutes	pH 1.2
60	80%

STI

	Relative Adjusted Volume from Densitometry (E:S*=10:1)
Minutes	pH 1.2
60	67%

* E:S refers to enzyme and substrate protein ratio at unit of pepsin per μg of substrate protein.
