# Supplementary material for: Analyzing pepsin degradation assay conditions used for allergenicity assessments to ensure that pepsin susceptible and pepsin resistant dietary proteins are distinguishable
Source: PLoS One. 2017 Feb 16;12(2):e0171926. doi: 10.1371/journal.pone.0171926 (PMC5312868; doi:10.1371/journal.pone.0171926)
Supplement: S4 File — (PDF) [file pone.0171926.s004.pdf]

## Supporting Information

**Fig 5. Comparison of pepsin degradation of five substrate proteins (HRP, Rubisco LS, Hb, STI, and LTP).** On Panel A and Panel B, the amount of Coomassie Blue stained intact protein at each condition was quantified and is shown as a percentage relative to the amount of starting material. Panel A is at 10 U pepsin to 1 μg substrate protein and pH 1.2. Panel B is at 10 U pepsin to 1 μg substrate protein and pH 4. Panels C and D are SDS-PAGE analysis of pepsin degradation of LTP and STI, respectively, at pH 1.2 and 10 U pepsin to 1 μg substrate protein over time (0.5 to 60 minute(s)). The gel lanes are: 1: MW, 2: Pepsin Only, 3: Substrate Protein Only, 4: 0 minute: Pepsin + Substrate Protein, 5 – 11: 0.5, 2, 5, 10, 20, 30, 60 minute(s), respectively, of Pepsin + Substrate Protein.

The average and standard deviation of relative adjusted volume from densitometric analysis for the three pepsin susceptible proteins (HRP, Rubisco LS, Hb) are listed below as well as the relative adjusted volume for the two pepsin resistant proteins (LTP and STI). The data were used to plot the line graphs.

HRP

| | Average of Relative Adjusted Volume from Densitometry (E:S*=10:1) | | STDEV of Relative Adjusted Volume from Densitometry (E:S*=10:1) | |
|---|---|---|---|---|
| Minutes | pH 1.2 | pH 4 | pH 1.2 | pH 4 |
| 0.5 | 1% | 98% | 1% | 1% |
| 2 | 1% | 98% | 0% | 3% |
| 5 | 0% | 96% | 0% | 2% |
| 10 | 0% | 97% | 0% | 1% |
| 20 | 0% | 98% | 0% | 3% |
| 30 | 0% | 97% | 0% | 3% |
| 60 | 0% | 96% | 0% | 1% |

Rubisco LS

| | Average of Relative Adjusted Volume from Densitometry (E:S*=10:1) | | STDEV of Relative Adjusted Volume from Densitometry (E:S*=10:1) | |
|---|---|---|---|---|
| Minutes | pH 1.2 | pH 4 | pH 1.2 | pH 4 |
| 0.5 | 1% | 48% | 1% | 13% |
| 2 | 1% | 23% | 0% | 16% |
| 5 | 1% | 9% | 0% | 10% |
| 10 | 1% | 3% | 0% | 3% |
| 20 | 1% | 2% | 0% | 1% |
| 30 | 1% | 1% | 0% | 0% |
| 60 | 1% | 1% | 0% | 1% |

Hb

| | Average of Relative Adjusted Volume from Densitometry (E:S*=10:1) | | STDEV of Relative Adjusted Volume from Densitometry (E:S*=10:1) | |
|---|---|---|---|---|
| Minutes | pH 1.2 | pH 4 | pH 1.2 | pH 4 |
| 0.5 | 1% | 47% | 1% | 11% |
| 2 | 0% | 36% | 0% | 14% |
| 5 | 0% | 24% | 0% | 16% |
| 10 | 0% | 12% | 0% | 11% |
| 20 | 0% | 6% | 0% | 6% |
| 30 | 0% | 3% | 0% | 3% |
| 60 | 0% | 1% | 0% | 1% |

LTP

| | Relative Adjusted Volume from Densitometry (E:S*=10:1) | |
|---|---|---|
| Minutes | pH1.2 | pH 4 |
| 0.5 | 90% | 92% |
| 2 | 79% | 93% |
| 5 | 82% | 91% |
| 10 | 85% | 91% |
| 20 | 78% | 105% |
| 30 | 75% | 102% |
| 60 | 80% | 94% |

STI

| | Relative Adjusted Volume from Densitometry (E:S*=10:1) | |
|---|---|---|
| Minutes | pH 1.2 | pH 4 |
| 0.5 | 104% | 95% |
| 2 | 102% | 100% |
| 5 | 105% | 103% |
| 10 | 101% | 105% |
| 20 | 94% | 105% |
| 30 | 84% | 101% |
| 60 | 67% | 98% |

* E:S refers to enzyme and substrate protein ratio at unit of pepsin per μg of substrate protein.
